# Analysing wideband absorbance immittance in normal and ears with otitis media with effusion using machine learning

**DOI:** 10.1038/s41598-021-89588-4

**Published:** 2021-05-20

**Authors:** Emad M. Grais, Xiaoya Wang, Jie Wang, Fei Zhao, Wen Jiang, Yuexin Cai, Lifang Zhang, Qingwen Lin, Haidi Yang

**Affiliations:** 1 Centre for Speech and Language Therapy and Hearing Science, School of Sport and Health Sciences, Cardiff Metropolitan University, Cardiff, CF5 2YB UK; 2 Department of Otolaryngology, Guangzhou Women and Children’s Medical Centre, Guangzhou City, Guangdong Province, 510623 China; 3 Department of Otolaryngology Head and Neck Surgery, Beijing Tongren Hospital, Beijing, 100730 China; 4 Key Laboratory of Otolaryngology Head and Neck Surgery, Ministry of Education, Beijing Engineering Research Centre of Hearing Technology, Beijing, 100730 China; 5 Department of Hearing and Speech Sciences, Xuzhou Medical University, Xuzhou City, Jiangsu Province, 221000 China; 6 Sun Yat-sen Memorial Hospital, Department of Otolaryngology, Sun Yat-sen University, Guangzhou City, Guangdong Province, 510120 China; 7 Institute of Hearing and Speech-Language Science, Sun Yat-sen University, Guangzhou City, Guangdong Province, 510120 China

**Keywords:** Health care, Medical research

## Abstract

Wideband Absorbance Immittance (WAI) has been available for more than a decade, however its clinical use still faces the challenges of limited understanding and poor interpretation of WAI results. This study aimed to develop Machine Learning (ML) tools to identify the WAI absorbance characteristics across different frequency-pressure regions in the normal middle ear and ears with otitis media with effusion (OME) to enable diagnosis of middle ear conditions automatically. Data analysis included pre-processing of the WAI data, statistical analysis and classification model development, and key regions extraction from the 2D frequency-pressure WAI images. The experimental results show that ML tools appear to hold great potential for the automated diagnosis of middle ear diseases from WAI data. The identified key regions in the WAI provide guidance to practitioners to better understand and interpret WAI data and offer the prospect of quick and accurate diagnostic decisions.

## Introduction

The human middle ear functions importantly for effective sound transmission by acting as an impedance matching device between the low impedance of air and high impedance of cochlear fluids^1^. Tympanometry is a useful tool for measurement of acoustic admittance changes in the middle ear system as air pressure varies in the external ear canal^2^. Conventional tympanometry with a single low-frequency, usually 220 or 226 Hz probe tone, is used routinely in audiological and otological assessment. Over the past five decades, a large body of research evidence has shown that tympanometry is an essential tool in the detection of certain types of middle ear pathology in ENT/Audiology clinics^2^. Technological advances in the assessments of middle ear function have expanded the frequency range from single probe tones (226/1000 Hz) to multiple frequency measurements delivered as a sweep through a series of frequencies^3^. This multiple frequency tympanometry (MFT) has been shown to provide improved sensitivity and specificity in the detection of some middle ear pathologies, such as otosclerosis and ossicular discontinuity^4^. In recent years a commercialised MFT device that uses Wideband Absorbance Immittance (WAI), also known as Wideband Energy Reflectance (WBER) or Otoreflectance has been developed^5^. This system is designed to assess wideband acoustic transfer function of the middle ear over a wide frequency range from 0.25 to 8.0 kHz^6^. The acoustic absorbance characterises the ratio of absorbed sound energy to incident sound energy. A number of studies have shown that measurement of absorbance has several advantages over traditional tympanometry^5,7^. Measurement of WAI is simple, fast, objective, reproducible and non-invasive, and because some changes of energy absorbance are associated with certain types of middle ear pathologies, the WAI has unique features that provide important diagnostic information in patients with middle ear disorders. Keefe et al.^8^ found that the likelihood-ratio predictors for wideband absorbance at ambient and tympanometric pressure was higher (0.97 to 0.93) than the predictors for conventional 226 Hz tympanometry (0.80 to 0.93) in the detection of conductive hearing loss. In addition Keefe and Simon^9^ showed that the sensitivity for diagnosing childhood otitis media with effusion (OME) increased from 27 to 78% when adding WAI measurement to the test battery thereby reducing inappropriate diagnosis and costs associated with the condition.

Recent studies have also proven the significant advantages of WAI in providing additional information on middle ear function by using a wider frequency range as a function of pressure, e.g., at ambient pressure and peak pressure, plotted in two- and three-dimensional graphs^6,10,11^. The multidimensional graphs obtained from WAI enable the clinician to better understand the dynamic characteristics of the middle ear by recognizing specific tympanometric patterns associated with middle ear pathologic change. Niemczyk et al.^12^ investigated WAI patterns in ears with intraoperatively confirmed otosclerosis by analysing resonance frequency, and number of peaks with detailed descriptions in terms of height and width. Although the patterns were statistically significantly different and provided important diagnostic information in terms of otosclerotic status, this approach appears less helpful clinically for the purpose of differential diagnosis as the absorbance patterns overlap with absorbance graphs found in the normal ear condition and other middle ear disorders. For example there is a similar characteristic of significantly reduced absorbance in frequencies below 2000 Hz in cases of OME^10^. Therefore, the main challenges clinicians still face are to understand, interpret and use WAI data as an effective and accurate diagnostic tool in ENT and Audiology clinics.

In addition, there is little research being undertaken to investigate the use of whole WAI data on energy absorbance, within which is embedded substantial information associated with energy transfer function of the middle ear, particularly in the high frequency region under various middle ear pressures^6,11^. A recent study by Hougaard et al.^11^ analysed the wideband energy absorbance (EA) tympanogram from 99 ears in normal middle ear conditions. The results revealed a trend of increasing EA in the lower frequencies as a function of frequency regardless of ear pressure. EA peaked at around 4.0–5.0 kHz under positive pressures between + 50 and + 150 daPa, followed by a sharp decrease at higher frequencies. Although this study provided important 3D absorbance information in adults with normal hearing and middle ear function, future studies are necessary to further understand the potential for WAI in clinical applications.

Machine learning (ML) tools have been used to explore and process different data to extract useful information, make predictions and inform decision making^13,14^. The initial motivation underlying this study was to address questions that have arisen from clinical challenges and fill in gaps in the literature, particularly in relation to the limited understanding and poor interpretation of WAI results across different pressures in various frequency regions. Therefore, we aimed to identify the characteristics of WAI absorbance across different frequency-pressure regions in normal middle ear conditions and ears with OME and to develop ML tools to automatically diagnose ears as normal or with OME. Initial statistical analysis compared absorbance values at different frequency-pressure regions in normal middle ears and middle ears with OME. This was followed by an evaluation of the performance of five ML models in classifying the WAI data as being from a normal middle ear or ear with OME. We also aimed to identify key regions in the WAI data that could guide the clinicians in deciding whether the WAI data was from a normal ear or ear with OME. To the best of our knowledge, the present study is the first to use ML tools to better understand and interpret WAI results, and provide automated diagnosis of ears with OME and thereby facilitate its clinical application.

## Materials and methods

### Materials

#### Wideband absorbance immittance (WAI) data acquisition

A total of 672 WAI data were collected from patients and volunteers in five hospitals in Beijing, Guangzhou and Xuzhou, China. There were 423 ears from 242 participants with normal middle ear function (age range 1–68), and 249 ears with OME from 163 participants (age range 1–73 years). The number of participants with bilateral OME was 86 (52.8%) and with unilateral OME was 77 (47.2%). Data from neonates and infants younger than 1 year old were excluded. Poor quality WAI measurements with incomplete pressure values were also excluded.

The definition and inclusion criteria for normal middle ear and ears with otitis media with effusion were:

- Normal middle ear function: (1) No history of inflammation or disease that has impacted the middle ear, nor any recent hearing disability and aural symptoms; (2) Otoscopy: normal tympanic membrane lustre normal, no atrophy, scar, retraction or perforation; (3) Tympanometry: normal range of middle ear pressures within − 50 to + 50 daPa (adults) or − 100 to + 50 daPa (children). Peak compliance range from 0.3 to 1.4 ml (adults) or 0.3 to 0.9 ml (children) (Type A tympanogram).
- Otitis media with effusion (OME): (1) OME is defined as fluid of varying amount and viscosity accumulated in the middle ear as a result of Eustachian tube dysfunction; (2) Otoscopy: tympanic membrane lustre is dull, either with air bubbles or a fluid line or retraction; (3) Tympanometry: abnormality of middle ear function measured and diagnosed by conventional tympanometry, showing (a) significant negative middle ear pressure in the presence of normal static compliance (Type C tympanogram), i.e., range of middle ear pressure is less than − 50 daPa (adults) or − 100 daPa (children), and range of peak compliance from 0.3 to 1.4 ml (adults) or 0.3 to 0.9 ml (children), or (b) no measureable middle ear pressure or static compliance (Type B tympanogram), i.e., a flat trace.

### Methods

The work included pre-processing of the WAI data, statistical analysis and ML model development, together with further key regions extraction from the 2D frequency-pressure WAI images.

#### WAI measuring system and data pre-processing

**Figure 1:**
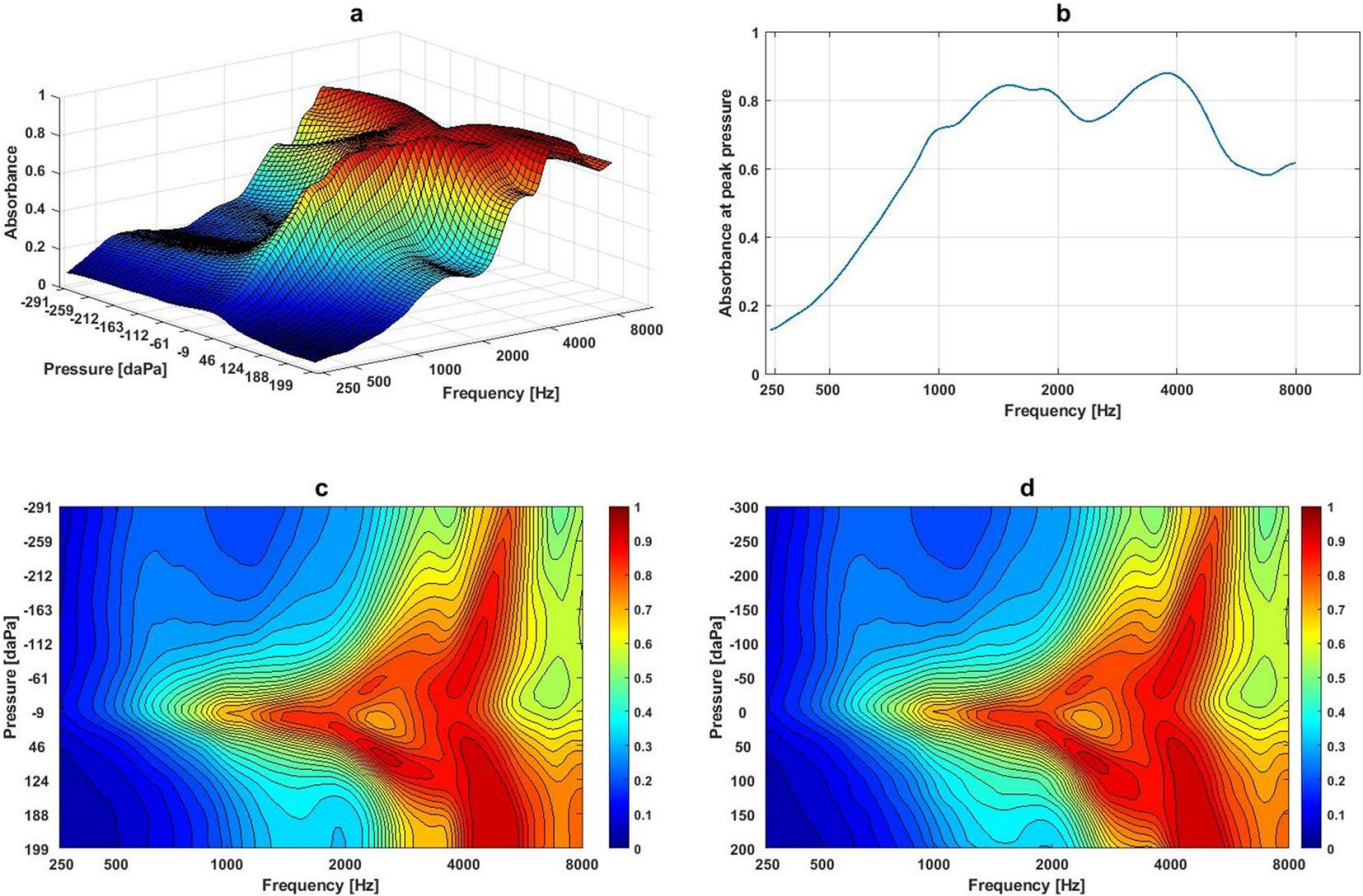
An example of the 3D WAI image and data pre-processing: (**a**) An example of 3D WAI data obtained from a participant with normal middle ear function aged 22; (**b**) the 2D frequency-absorbance plot at the peak pressure obtained from same participant; (**c**) the 2D frequency-pressure image converted from the 3D WAI in Fig. 1a; (**d**) the 2D frequency-pressure image converted from the 3D WAI in (**a**) after interpolating the pressure values on the Y-axis.

A Titan IMP440 (Interacoustics, Denmark) was used to measure the 3D wideband absorbance. Figure 1a shows an example of a 3D image of Wideband Absorbance Immittance (WAI) obtained from a participant with normal middle ear function aged 22 years. The figure shows one dimension of the WAI to be frequency, the second pressure, and the third absorbance value^6^. Frequencies varied from 226 to 8000 Hz with 1/24-octave frequency-intervals. Pressures varied from − 300 to + 200 daPa, and absorbance was between zero and one. Theoretically, higher absorbance indicates a better transfer function of the middle ear, whereas lower absorbance means less energy being passed through the middle ear, indicating pathological change in the middle ear. Figure 1b shows the absorbance curve at peak pressure across a wide frequency range currently used to evaluate middle ear function. Figure 1c shows a 2D image using the domains of frequency and pressure corresponding to the WAI data in Fig. 1a. Values of absorbance show as values of pixels in the image. There are 107 bins across the frequency axis (X-axis) starting at 226 Hz and rising to 8000 Hz. Because the pressure axis was unevenly sampled due to artefact rejection of noisy samples, ear canal volume, and probe fit^11^, we resampled the pressure axis between − 300 to + 200 daPa in 10-daPa steps using the Piecewise Cubic Hermite Interpolating Polynomial^15^. As a result, there are 51 pressure values on the Y-axis as shown in Fig. 1d. There were a total of 5457 data points, i.e., 107-frequency bins × 51-pressure values in the 2D frequency-pressure WAI images.

#### Statistical analysis

Mean and variance of absorbance at each frequency-pressure region was calculated for participants with normal middle ear function and those with OME. A Wilcoxon rank sum test was used to determine any significant differences between the normal and OME ears. The level of significance was set at the conventional 5% level.

#### Development of machine learning (ML) models for WAI classification

Basic ML classifiers were used to process the 2D WAI images after interpolating the pressure axis; K-nearest neighbours classifier, support vector machines (SVM), and random forest (RF). Deep learning (DL) based classifiers, such as feedforward neural networks (FNN) and convolutional neural networks (CNN) were also examined. The ultimate goal of developing ML classifiers was to enable the automatic classification of WAI data as being from normal or OME ears.

#### Extracting the important regions from 2D WAI images

In clinical practice, as a part of clinical assessment, medical images (e.g., X-ray, CT scan and MRI) play an important role to detect and identify the pathological changes, and thus facilitate clinical diagnosis. Experienced clinicians are usually aware of the key regions to be examined in those images, and consequently achieve a quick and accurate judgement that leads to an appropriate diagnostic decisions. In this study, two feature extraction techniques were used to identify the important regions from 2D WAI images, i.e., (1) random forest classifiers as a feature selection tool to extract the important features of the 2D images, and (2) significance tests over the 2D frequency-pressure WAI images to extract regions showing the most significant differences between normal ears and OME ears. The extracted regions could provide valuable guidance to audiologists and ENT physicians in their diagnostic decisions.

### Ethics

All methods used in this study were approved by Cardiff School of Sport and Health Sciences Ethical Committee under the Cardiff Metropolitan University ethical guidelines and regulations (Ethical reference number: Sta-3013). Informed consent was obtained from all subjects, and if subjects were under 18, from a parent and/or legal guardian. The anonymous WAI data were analysed when machine learning tools were used.

## Results

### WAI characteristics and statistical analysis of the normal middle ear condition compared to ears with OME

**Figure 2:**
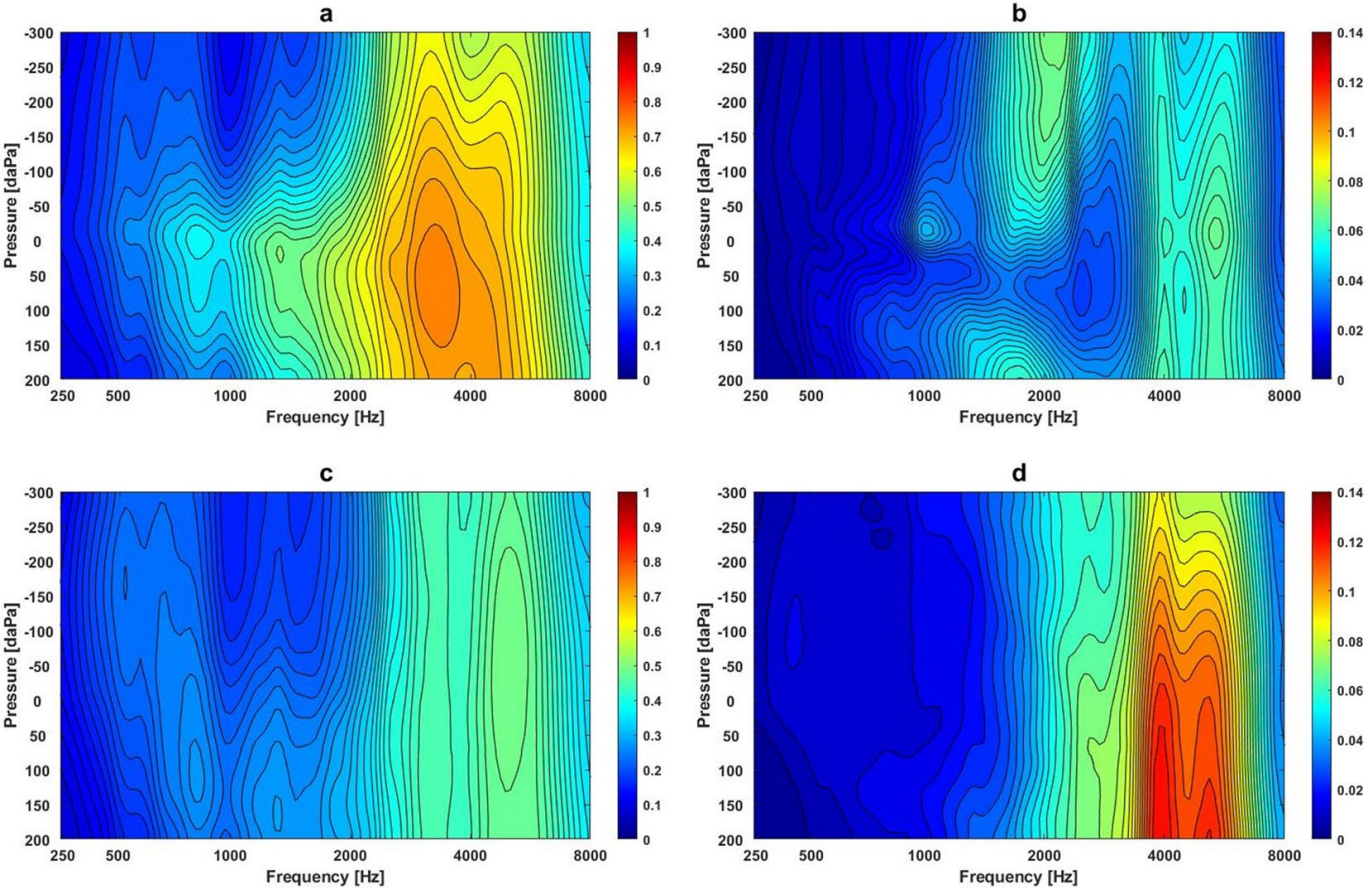
The mean and variance in absorbance at different frequency-pressure regions in the normal middle ear condition and ears with OME: (**a**) Mean absorbance contour plot at different frequencies and pressures in the normal middle ear condition; (**b**) Variance of absorbance contour plot at different frequencies and pressures in the normal middle ear condition; (**c**) Mean of absorbance contour plot at different frequencies and pressures in ears with OME; (**d**) Variance of absorbance contour plot at different frequencies and pressures in ears with OME.

Figures 2a–d show the mean and variance of absorbance at different frequencies and pressures in the normal middle ear and ears with OME. The averaged absorbance contour for the normal middle ear condition showed a peak area at the centre frequency of 820 Hz at 0 daPa with an absorbance value of 0.39 (frequency range: 771–917 Hz with pressures between − 30 and + 30 daPa and absorbance value 0.4), and the second peak at the centre frequency of 1335 Hz at + 20 daPa with absorbance value of 0.50 (frequency range: 1300–1370 Hz with pressures between 0 and + 40 daPa and absorbance value 0.5). The largest peak occurred at the centre frequency 3270 Hz at + 65 daPa, with absorbance at 0.76) (frequencies range: 2900–3700 Hz with pressures between − 30 and + 160 daPa and absorbance values between 0.75 and 0.76 (Fig. 2a). Figure 2b shows the contour of variance for the normal middle ear condition. There were a couple of areas showing larger variances in absorbance obtained from normal middle ears, i.e., variance value of 0.07 at frequencies between 1834 and 2370 Hz at pressures between − 300 and − 110 daPa, and the variance value of 0.07 at frequencies between 5180 and 5500 Hz at pressures between − 30 and + 10 daPa. In comparison, ears with OME showed the largest peak centred at 5000 Hz with pressure at − 30 daPa with an absorbance value of 0.5 (frequency range: 4500 to 5500 Hz, pressures from − 220 to + 130 daPa with an absorbance value of 0.5) (Fig. 2c). The highest value in the averaged absorbance was significantly lower in the OME ears than the normal ears (0.49 vs. 0.76, *p* < 0.0005). Significantly higher variances in absorbance were found in OME ears, with the largest averaged variance occurring between 3700 and 5500 Hz and at pressures between + 40 and + 200 daPa and variance values from 0.11 to 0.12 (Fig. 2d). Further statistical analysis at each frequency-pressure point showed 88% of data points (4782 out of 5457) to have significant differences in absorbance values between normal ears and OME ears (Wilcoxon rank sum test, *Z* = 3.04, *p* < 0.0005).

### Outputs from various classification models: accuracy, area under the ROC curve, F1 score, precision and recall for categorising middle ear function as normal or OME

As indicated in section “Methods”, different ML classifiers were developed and examined to determine their ability to categorise middle ear function as normal or OME using the 2D frequency-pressure WAI images. For the KNN, SVM, RF, and FNN classifiers, the 2D images were converted into vectors with the dimension 5457, i.e., 107-frequency bins × 51-pressure values. The 2D images were used directly for the CNN. Data were normalized to have a zero mean and unit variance for all classifiers. We used the 10-fold cross validation to test each model. There is some randomization in the process of RF and in the initialization of FNN and CNN and since the performance of these classifiers depends strongly on this initialization, especially with small datasets, we ran the experiments three times with different random initializations for each model run. To implement the neural networks, Keras with TensorFlow backend was used^16^ and for the remaining classifiers, we used Scikit-learn Python based library^17^. Table 1 summarises the results derived from each ML model in terms of; accuracy, area under the ROC curve, precision, recall/sensitivity and F1-score for predicting normal from OME^13^. Overall, the different ML models produced different outputs. The area under the ROC curve ranged from 0.74 to 0.79, while the accuracy for determining the middle ear function ranged from 0.74 to 0.82. Of these, the CNNs had the highest accuracy as the most promising models. We tested different KNN classifiers with different number of neighbours in queries, i.e., $K=1, 3, 15$. All points in each neighbourhood were weighted equally and Euclidean distance was used to measure the distance between data samples. The results of KNN shown in Table 1 indicate that increasing *K* improves the accuracy and precision for OME, the recall for the normal and F1-scores for both classes, but decreases the recall of the OME and slightly decreases the precision for the normal cases. This indicates that with increasing *K* the model is biased toward classifying the data into normal and this perhaps because the number of normal samples is higher than the number of OME samples in our dataset. For SVM, different kernels were examined, such as, linear, polynomial (Poly), radial basis function (RBF), and sigmoid^13^. The degree for the polynomial kernel was three. As shown in Table 1, the accuracy and F1-scores, for the polynomial and RBF kernels were better than the other kernels. For the RF, different numbers of decision trees were tested in the forest combination, e.g., 10, 100, 500. The RF was run three times with different randomization for each run. As shown in Table 1, the best results (accuracy, F1-scores, precision, and recalls for both classes) were obtained using 100 and 500 decision trees in the RF. With the experiments using Feedforward neural networks (FNN), different number of layers and different number of nodes in each layer were developed and examined. The detailed structure of each FNN is described in Table 2. The rectified linear unit (ReLU) was used as an activation function in the hidden layers and the sigmoid activation function was used in the output layer. A dropout value 20% was used after each hidden layer. The tested FNNs gave almost the same results as shown in Table 1. Convolutional neural networks (CNN) experiments also used a different number of layers and different number of filters and different filter sizes in each layer. The detailed structure of each layer in each CNN is described in Table 2. Similar values were found in terms of the accuracy and F1-score in all CNNs, together with small differences in the precision and recalls for both classes using CNNs with different structures as shown in Table 2. To train the FNN and CNN models, the binary cross entropy cost function and Adam optimizer were used. Because the initialization of the FNNs and CNNs is important, each model was trained three times with different random initialization for each training, i.e., training three models with the same structure but with different initializations.

**Table 1:** Summary of the performance of the ML models for predicting normal middle ear condition and OME.

Classifier	Design	AUC–ROC	Precision		Recall		F1-score		Accuracy
			Normal	OME	Normal	OME	Normal	OME	
KNN	1	0.74	0.83	0.64	0.75	0.73	0.79	0.68	0.75
	3	0.75	0.81	0.68	0.81	0.69	0.81	0.68	0.76
	15	0.78	0.81	0.80	0.91	0.65	0.86	0.72	0.81
SVM	Linear	0.74	0.83	0.63	0.75	0.73	0.79	0.68	0.74
	Poly	0.77	0.81	0.77	0.89	0.65	0.85	0.71	0.80
	RBF	0.77	0.80	0.81	0.92	0.62	0.86	0.70	0.80
	Sigmoid	0.74	0.79	0.72	0.86	0.62	0.82	0.66	0.77
RF	10	0.77	0.81	0.75	0.87	0.66	0.84	0.70	0.79
	100	0.78	0.83	0.76	0.87	0.69	0.85	0.72	0.80
	500	0.78	0.83	0.76	0.87	0.69	0.85	0.72	0.80
FNN	FNN1	0.78	0.83	0.77	0.88	0.69	0.85	0.73	0.81
	FNN2	0.79	0.83	0.78	0.89	0.69	0.86	0.73	0.81
CNN	CNN1	0.79	0.83	0.79	0.90	0.69	0.86	0.74	**0.82**
	CNN2	0.79	0.83	0.78	0.89	0.70	0.86	0.74	**0.82**

**Table 2:** The structures for the FNN and CNN models.

Layers/models	FNN1	FNN2	CNN1	CNN2
1	Dense(1000)	Dense(1000)	Conv2D(20, (21, 11))	Conv2D(20, (21, 11))
			MaxPooling2D(3,2)	MaxPooling2D(3,2)
	Activation(‘relu’)	Activation(‘relu’)	BatchNormalization	BatchNormalization
			Activation(‘relu’)	Activation(‘relu’)
			Dropout(0.2)	Dropout(0.2)
2	Dense(100)	Dense(500)	Flatten	Conv2D(40, (11, 7))
				BatchNormalization
	Activation(‘relu’)	Activation(‘relu’)		Activation(‘relu’)
				Dropout(0.2)
3	Dense(1)	Dense(100)	Dense(100)	Conv2D(60, (3, 3))
			Activation(‘relu’)	BatchNormalization
	Activation(‘sigmoid’)	Activation(‘relu’)	Dropout(0.2)	Activation(‘relu’)
				Dropout(0.2)
4	–	Dense(1)	Dense(1)	Flatten
		Activation(‘sigmoid’)	Activation(‘sigmoid’)	
5	–	–	–	Dense(100)
				Activation(‘relu’)
				Dropout(0.25)
6	–	–	–	Dense(1)
				Activation(‘sigmoid’)
Number of parameters	5,558,201	6,008,701	1,754,921	5,338,621

The symbol “–” means the layer does not exist and Dense(1000) means fully connected layer with 1000 nodes. Conv2D(20, (21, 11)) means a 2D convolutional layer with 20 filters, where the size of each filter is 21 in the frequency direction and 11 in the pressure direction. MaxPooling2D (3,2), is a 2D max-polling operator with size 3 in the frequency direction and 2 in the pressure direction. Dropout (0.2) means 20% dropout. ’relu’ means rectified linear unit activation function.

### Handling imbalanced datasets and the performance of the CNN models

**Figure 3:**
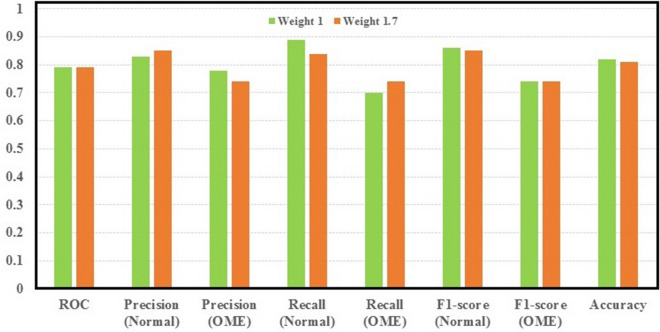
Performance of CNN2 with using different weights on the OME samples in the cost function.

As the dataset used in this study includes 423 samples from normal class and 249 samples from the OME class, i.e., the normal class samples are 1.7 times the OME samples, imbalance issue between the two classes may lead the classifiers to perform better with the majority class than the minority class^13,18^. This can be fixed using different methods, such as, resampling data space and cost-sensitive learning^18^. In this study, a succinct approach based on cost-sensitive learning was used to penalize the errors arising from the misclassification of the minority class more than the error coming from the majority class during training the models. This was achieved by putting more weight on the errors from the misclassification of the minority class in the cost function for training the models^18^. Consequently, the error coming from the misclassification of the OME sample was weighted 1.7 times to the error coming from the misclassification of the samples from the normal middle ear class during training of the CNN models. The influence of the weighting rates was examined using the CNN2 model as shown in Table 2. Figure 3 summarises the results obtained from using different weights to fix the imbalanced issue between normal and OME samples. The results showed an increase in the recall of the OME by 5%, when we penalized the misclassification of the OME class by increasing the weight to 1.7. It implies that there is the possibility of improving predictive performance for OME cases by fixing the imbalance issue of the dataset.

### Extracting the discriminative regions of the 2D frequency-pressure WAI images

In this experiment, ML tools were used as data driven approaches to extract the discriminative regions from the WAI, which would provide useful guidance for clinicians in their diagnostic decisions. Two different data driven approaches were investigated:-

1. The first approach was based on extracting the key regions that derive the random forest (RF) classifier to categorize the WAI as normal or OME. In this approach, the RF classifier was used as a feature selection approach to extract the key regions from the WAI;
2. The second approach was to extract the regions in the 2D WAI images that indicate significant difference values between the classes using a Wilcoxon rank sum test^19^.

#### Using random forest classifiers to extract the discriminative regions in the 2D WAI images

**Figure 4:**
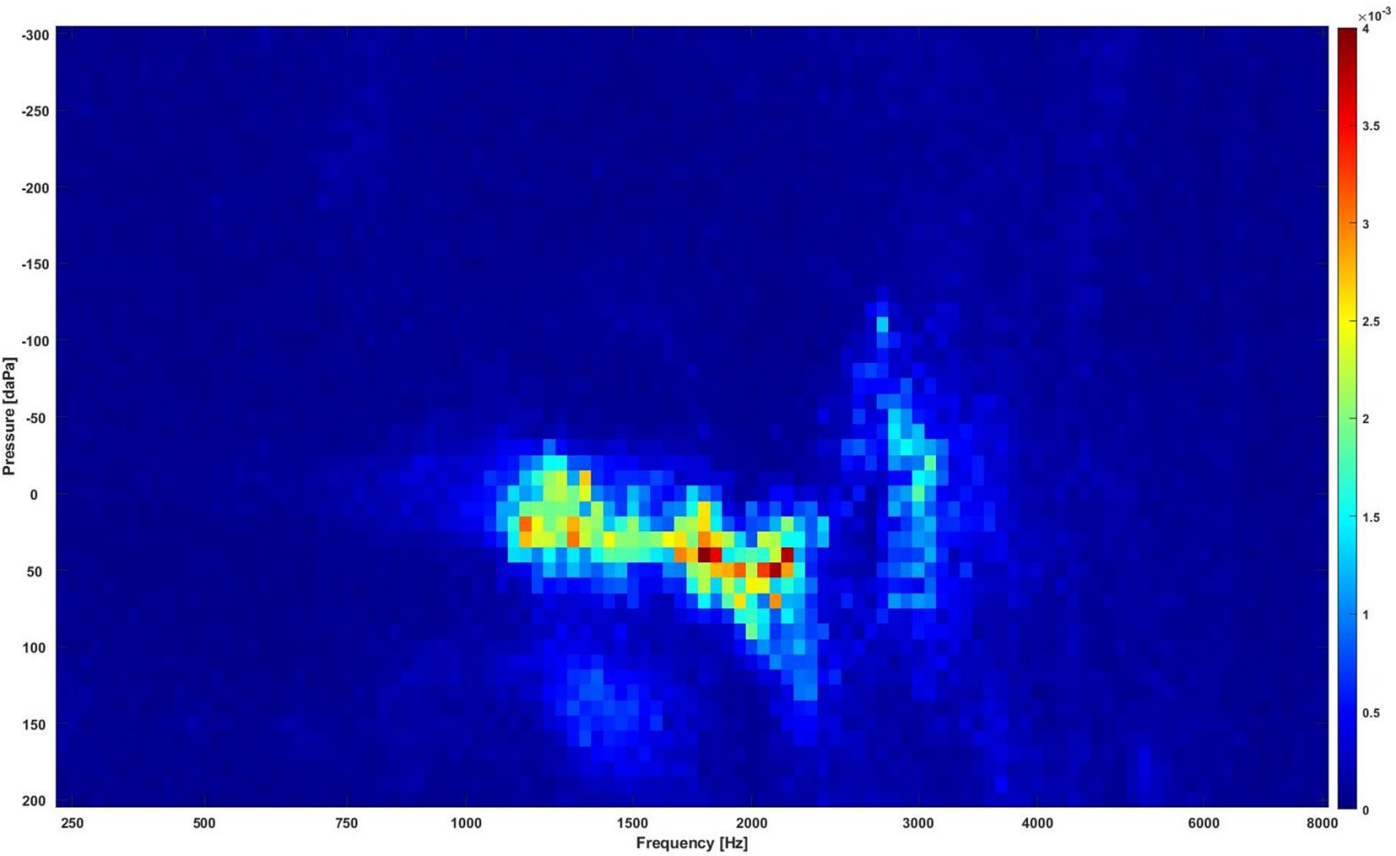
The extracted discriminative regions in the WAI data using the RF classifiers. Regions with high values (bright regions) are the most discriminative regions.

The RF based feature selection technique extracts regions that mostly drive the classification decision by giving the important features that carry the most discriminative information more values than redundant features. Ten different RF with a different number of decision trees were tested, i.e., 10, 20, 30, 40, 50, 100, 200, 300, 400, and 500 trees. In each RF case, a set of coefficients were obtained representing the importance of the features in the 2D images. The coefficients from the ten RFs were then averaged to achieve a smoother estimate for the extracted regions. Figure 4 shows the averaged coefficients from the ten RF cases. The highlighted regions with high values indicate discriminative regions for the different classes. There was an important discriminative region with high values around frequencies from 1000 to 2670 Hz and pressures from − 50 to + 100 daPa that showed a significant difference between normal and OME data.

#### Using statistical significance tests to extract discriminative regions in the 2D WAI images

**Figure 5:**
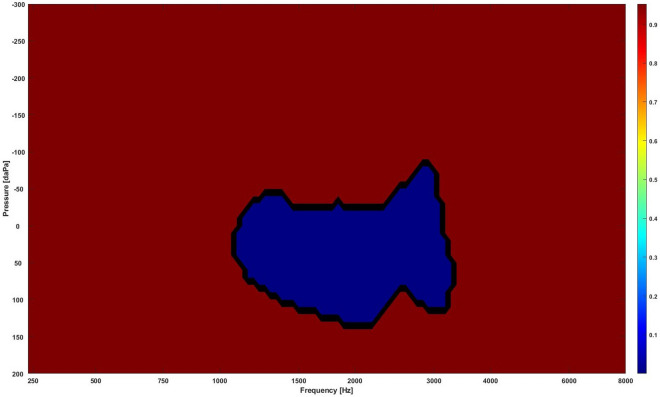
The most significantly different region (blue) in the WAI data. The blue region is the region with the lowest *p*-values that contains 10% of the data ($51*107*10\% = 546$ points).

Section “WAI characteristics and statistical analysis of the normal middle ear condition compared to ears with OME” showed that 88% of the total area in the 2D frequency-pressure images indicate significant difference in absorbance between normal and OME ears. In this experiment, the top 10%, i.e., 5457 points 10% = 546 points) of the most significantly different points, i.e., the lowest *p*-values between the two classes in the 2D frequency-pressure images were highlighted as shown in Fig. 5.

**Figure 6:**
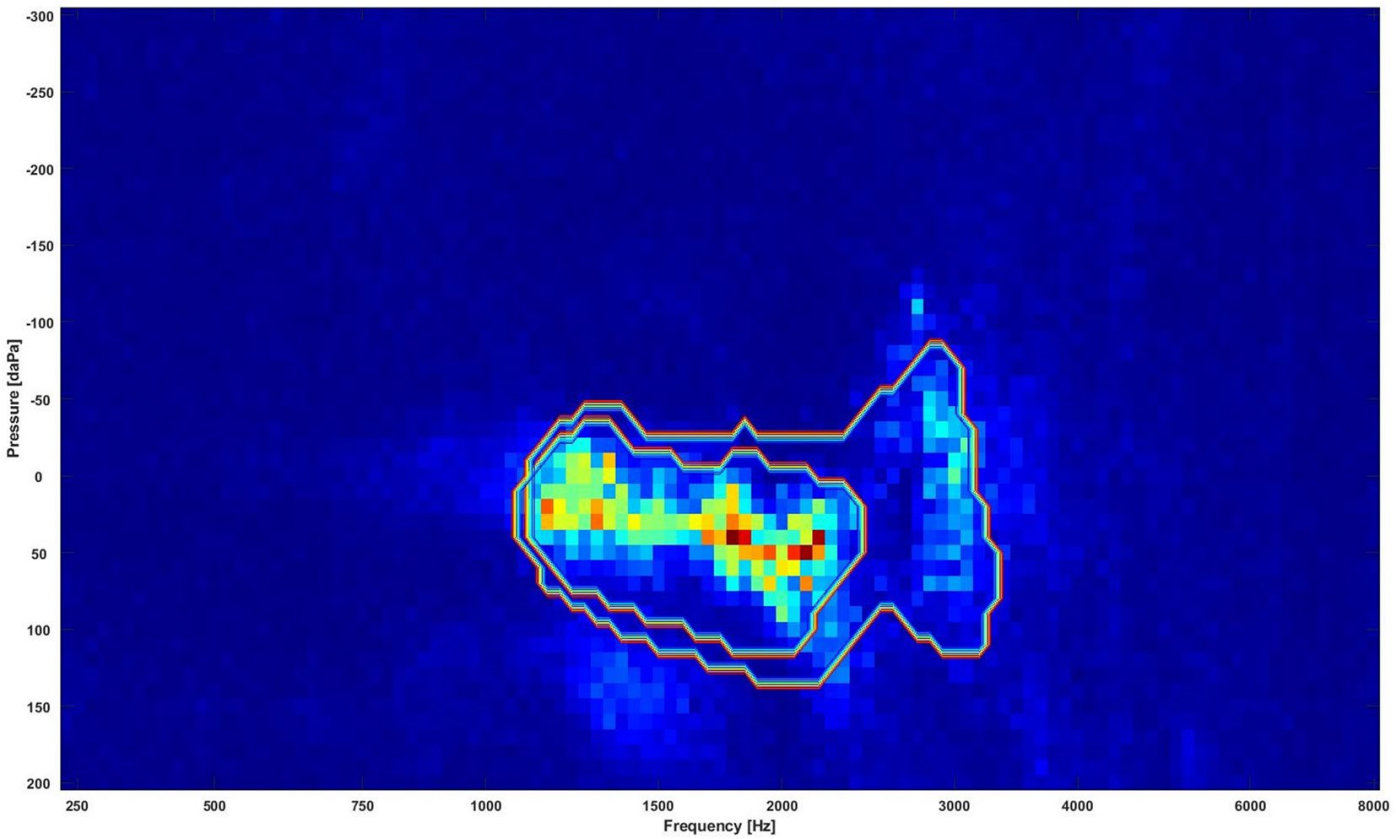
The combination of the regions extracted by RF and contours of extracted regions by the significance test, where the inner contour contains the 5% most significant points (273 points) and the outer contour the top 10% most significant points. Bright regions are the key regions extracted by the RF. The inner circle highlights the statistically significant area that contains 273 points with the lowest *p* values (273/5457, 5.0%). The outer circle features the statistically significant area that is comprised of points with the 546 lowest *p* values (546/5457, 10.0%).

Figure 6 shows regions extracted from RF with extracted region contours identified using the statistical significance test. The inner contour contains 5% of the most significantly different points (273 points), the outer contour contains the top 10% of the most significantly different points. Figure 6 demonstrates that the RF and the significance test approaches picked almost the same regions. Further analysis showed the averaged absorbance in the 10% extracted region was 0.59 and 0.33 for normal and OME ears respectively. For the 5% extracted region mean absorbance was 0.53 for normal and 0.28 for OME ears.

## Discussion and future study

According to the recent ENT Elective Care Handbook, the biggest challenge for ENT specialists appears to be ‘unnecessary’ referrals by GPs that could be managed effectively in primary care. An updated Clinical Practice Guideline on Otitis Media with Effusion^20^ points out that a low percentage of clinicians follow clinical practice guidelines in the use of pneumatic otoscopy for diagnosis due to a lack of experience in handling the technical difficulties of the device. Consequently efforts are still needed in primary care settings to teach and promote accurate OME diagnosis.

In the present study, several ML classifiers have been examined using different parameters for the purposes of categorising middle ear function as normal or OME on the basis of WAI data. As shown in Table 1, results from most of the tested classifiers are promising with the accuracy of most of the classifiers at around 80%. Indeed, the results from the ML classifiers in this study exceed diagnostic performance in identifying normal and OME ear conditions in the primary care settings by General Practitioners (GP) or other healthcare professionals using traditional middle ear diagnostic tools^10^. A study by Lee et al.^21^ investigated the accuracy of traditional diagnostic tools for OME, such as pneumatic otoscopy, otomicroscopy, and tympanometry. Their results showed low specificity in diagnosing childhood OME, although pneumatic otoscopy is recommended as the gold standard for OME diagnosis. In addition, there were high percentages of false positive and false negative cases when the results obtained from traditional tympanometry were examined. Future research will focus on improving the performance of the CNNs in terms of achieving more accurate and reliable classification results. Performance could be improved by using either larger datasets or advanced DL techniques such as transfer learning, data augmentation, and few-shot learning^22^ to train the CNNs more efficiently with small datasets. The work by Feyjie et al.^23^ demonstrates the efficiency of using few-shot learning in the task of skin lesion segmentation. A review on the state-of-the-art data augmentation methods applied in the context of segmenting brain tumours from MRI images indicates that data augmentation has become a main part of almost all DL methods for segmenting brain lesions^24^. Moreover, transfer learning has become a useful approach for analysing medical imaging using DL^25^.

This is the first study to use ML models to better understand and interpret the clinical meanings of characterized WAI regions that are closely associated with middle ear transfer function and further facilitate its clinical application. Two feature selection methods (i.e., random forest and statistical significance tests) were used in this study to extract the key regions from the WAI. The extracted regions could guide the clinicians to decide whether the case is normal or OME. Lai et al.^26^ investigated three other feature selection methods to extract dominant features to distinguish cases with temporal lobe epilepsy (TLE) from healthy cases. They were independent sample *t*-test filtering, the sparse-constrained dimensionality reduction model (SCDRM), and the support vector machine-recursive feature elimination (SVM-RFE). By using Support vector machine (SVM) to determine abnormal brain regions in TLE, their results indicated that the SVM-RFE achieved the best results, followed by the SCDRM and the *t*-test. More advanced DL tools such as attention mechanisms could also be used to extract the key regions in our future studies^27^. With attention mechanism, the neural network can weight features by level of importance to the classification task, and use this weighting to help achieve classification with better accuracy. Guan et al.^28^ showed that the performance of automated classification of thorax disease on the basis of chest X-ray images using attention mechanisms was improved in terms of accuracy by cropping out the discriminative parts of the image and classifying both the global image as well as the cropped portion together. In the present study, the size of the key region extracted and driving the classification decision is approximately 5% of the whole WAI image (as shown in Fig. 4), i.e., around frequencies from 1000 to 2670 Hz and pressure from − 50 to + 100 daPa, approximately 5% of the whole WAI images. This result provides important guidance to ENT physicians, audiologists and other healthcare professionals in terms of WAI data interpretations and subsequent diagnostic process for identifying middle ear diseases in the clinical setting. The small size of the key regions suggests that dimensionality reduction techniques could be used before classification to decrease the size of the data, allowing efficient computing, simplifying the complexity of the problem and possible improvement of results^29^. The study by Zhao et al.^7^ analysed the characteristics of 2D WAI plot configurations in ears with normal middle ear function. The results highlighted that the frequency region with high absorbance; 1.1 kHz (SD: 0.3 kHz appeared related to resonances in the middle ear system, where sound energy coming into the external ear canal is transmitted most efficiently into the cochlea^30^. A previous study by Beers et al.^31^ found that the area of the ROC curve was 0.9 at frequencies between 800 Hz and 5.0 kHz, with the best result at 1.25 kHz. 96% sensitivity and 95% specificity were achieved at the absorbance cut-off value of 71.7% in diagnosing childhood OME with WAI. Their results also imply the importance of areas around the middle ear resonance frequency. Furthermore, Zhao et al.^7^ found another region with high absorbance in the high frequency region (mean: 3.4 kHz, SD: 1.5 kHz). They suggested that this region might be associated with the external ear canal resonance and middle ear structure. A recent study by Won et al.^32^ concluded that the otitis media group with high viscosity effusion had significantly less absorbance from 2.74 to 4.73 kHz in comparison to the otitis media group with low viscosity effusion. In addition, the amount of middle ear effusion affected the absorbance at the frequencies from 1.92 to 2.37 kHz. However, their results did not show the statistical significance in absorbance around 1.0 to 2.0 kHz with effusion categories. This is likely due to the large variations in middle ear pressure that affect absorbance. Zhang and Gan^33^ investigated the effect of the middle ear pressure on WAI using the Finite Element (FE) model that simulated the negative middle ear pressure levels between − 50 and − 200 daPa. Their results showed that absorbance decreased in general with increased negative middle ear pressure, with the greater affected EA at frequencies between 1.0 and 4.0 kHz. Therefore, the impact of OME conditions defined using different classifications should be further investigated to compare the accuracy in ears with various OME severity.

Because of the complexity of 3D measurement results obtained from WAI, very few have explored the ability of WAI to differentiate between normal middle ears and OME, although a pilot study by Wang et al.^10^ investigated the dynamic characteristics of the middle ear system using 3D image analysis in ears with normal middle ear function and in the OME condition. They reported that the areas in the frequency range between 1.0 and 8.0 kHz with normal middle ear pressure appeared important in terms of distinguishing normal from OME. Absorbance in the high frequency region under high positive pressure was significantly decreased in ears with OME. In the present study, the contour of averaged absorbance in the frequency-pressure plot in normal ears is generally consistent with the findings of Hougaard et al.^11^. The averaged absorbance increases from 50% at 1.0 kHz to an absorbance peak point around 75% at 3.5 kHz under positive pressures between + 50 and + 150 daPa, followed by a sharp decrease at higher frequencies (Fig. 2a). Averaged absorbance in ears with OME were significantly lower than those in normal ear conditions (Fig. 2c). In comparison to the variance found in the normal ear conditions (Fig. 2b), significantly higher variances were found in absorbance in ears with OME around the frequency from 4.0 to 6.0 kHz in the positive pressure region (Fig. 2d). Won et al.^32^ also identified large variance in absorbance between 2.0 and 5.0 kHz in ears with OME of various type and amount of effusion. In another theoretical analysis using the FE model of the middle ear, Koike and Wada^34^ suggested that positive pressure in the middle ear cavity had a greater impact on sound transmission than negative pressure at frequencies beyond 1.5 kHz. Therefore, the area with greater variance at the region of high frequency and positive middle ear pressure should be used as an indicator of severity in the OME condition.

This novel research proves the capability of ML tools to diagnose OME automatically with an accuracy around 75–82%. Although the current accuracy might not sound perfect, the promising outcomes and ML solution provide an important stepping stone to inspire more researchers to work on this challenge and thus further facilitate its clinical application. Moreover, in applying ML to complex tasks in the diagnosis of OME, the technology has great potential for the development of a quick, accurate, cost effective, non-specialist diagnostic tool, with the potential for widespread use in global hearing healthcare to satisfy urgent clinical need.

It should be noted that there are several limitations in the present study. First, the sensitivity and specificity in the diagnosis of OME might be improved if a different gold standard is used (e.g., pneumatic-otoscopy, otomicroscopy or surgical confirmation of OME at time of ventilation tube insertion)^20^. However, whilst a myringotomy can serve as the gold standard for classifying an ear with OME, such a surgical procedure is invasive, and cannot be used to diagnose the presence of OME in patients with mild symptoms of middle-ear dysfunction. Although current clinical practice guidelines to diagnose OME primarily recommend pneumatic otoscopy, this examination is usually performed by an otologist who has a specialist training and experience on a visual inspection of tympanic-membrane mobility in response to pressure changes in a sealed ear canal. Second, in the present study, although we collected data from the normal middle ear condition and ears with OME from both children and adults, we are unlikely to be able to train a reliable machine learning model by dividing the current data into various age groups, due to limited sample size. Therefore, with a larger dataset, further analysis will be conducted to compare the accuracy across different age groups with various OME severity.

## Conclusion

In this work, the accuracy and the area under the ROC curve obtained from the basic ML models were around 75% and 80%, respectively. The convolutional neural networks show slightly better results than the other models. The promising results from this study indicate that the ML approach is a useful tool to help the non-specialist healthcare practitioner in providing an effective and accurate method for the automated diagnosis of OME. A region around frequencies between 1090 to 2310 Hz and pressures from − 40 to + 90 daPa extracted from the WAI by the RF classifiers and the statistical significance tests indicates important areas to identify differences between normal and ears with OME. The significance of the results provide clear guidance to practitioners to better understand and interpret the WAI data, and further facilitate its clinical applications. Future studies will focus on analyzing more WAI data on various middle ear disorders, e.g., otosclerosis, chronic otitis media and tympanic membrane perforation, using more robust DL tools.

## Data Availability

No additional data are available. However, the original data that support the findings derived from this study can be requested by emailing fzhao@cardiffmet.ac.uk.
